# Benign multicystic mesothelioma of peritoneum complicating acute appendicitis in a man: a case report

**DOI:** 10.1186/s13256-016-0826-6

**Published:** 2016-02-27

**Authors:** Savino Occhionorelli, Daniela Tartarini, Giovanni Pascale, Stefano Maccatrozzo, Rocco Stano, Giorgio Vasquez

**Affiliations:** 1 Department of Morphology, Surgery and Experimental Medicine, University of Ferrara, via Luigi Borsari 46, 44121 Ferrara, Italy; 2 Department of Surgery, Emergency Surgery Service, Arcispedale Sant’Anna, via Aldo Moro 8, Cona, 44124 Ferrara, Italy

**Keywords:** Acute appendicitis, Multicystic mesothelioma, Peritoneal neoplasm

## Abstract

**Background:**

Benign multicystic mesothelioma is a rare pathology. Few cases are reported in the medical literature and acute presentation is extremely uncommon.

**Case presentation:**

We describe an acute clinical presentation of the neoplasm that revealed itself with signs and symptoms attributable to acute appendicitis in a 41-year-old white man. Abdominal echography and computed tomography scans demonstrated the presence of a mass in direct contiguity with cecal fundus, but diagnosis remained unclear. Our patient underwent surgery and complete removal of the neoplasm. Only a definitive histological examination defined the nature of the lesion. No signs of relapse were demonstrated 1 year after the operation.

**Conclusions:**

We showed that an acute presentation of a benign neoplasm represents a diagnostic and therapeutic challenge for the surgeon, because of the difficult differential diagnosis that acute presentation can sometimes pose and the trouble that an emergence treatment can imply.

## Background

Benign multicystic mesothelioma is a rare pathology involving peritoneal serosa. We present the case of a benign peritoneal mesothelioma affecting cecum and pericecal peritoneum, complicating acute appendicitis in a man. In the international literature few cases are reported about this kind of neoplasm, and an acute presentation, as occurred in our case, is extremely rare [1]. Our patient underwent complete removal of the neoplasm and at 1-year computed tomography (CT) follow-up no evidence of pathology remained.

## Case presentation

**Fig. 1:**
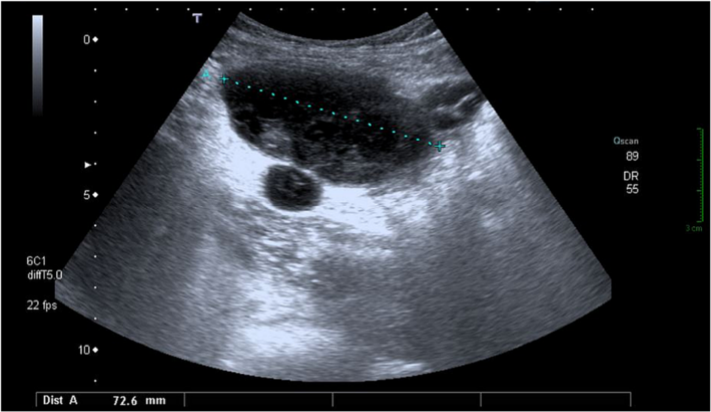
Abdominal ultrasonography. Evidence of an oval lump of 72 mm diameter and two less voluminous areas with hyperechogenic structure and hypoechoic-anechoic areas and septa in its contest

A 41-year-old white man was admitted to our Emergence Surgery Department with a diagnosis of acute appendicitis and probable saccate peritonitis. He presented with a history of abdominal pain of 1 week which was localized prevalently in his right iliac fossa. At admission he was subpyretic. A clinical examination showed slight pain to abdominal palpation at right iliac fossa and flank, where a mass was appreciable, without signs of peritonism; peristalsis was preserved. Laboratory examinations showed little enhancement of inflammatory indexes (PCR 2.10 mg/dL, fibrinogen 471 mg/dL) without leucocytosis or any other relevant alteration. An abdominal ultrasonography was performed; it revealed evidence of an oval lump of 72 mm diameter with hyperechogenic structure and hypoechoic-anechoic areas and septa in its contest, near to two similar less voluminous areas (Fig. 1). This report was erroneously interpreted as a zone of saccate peritonitis complicating acute appendicitis.

**Fig. 2:**
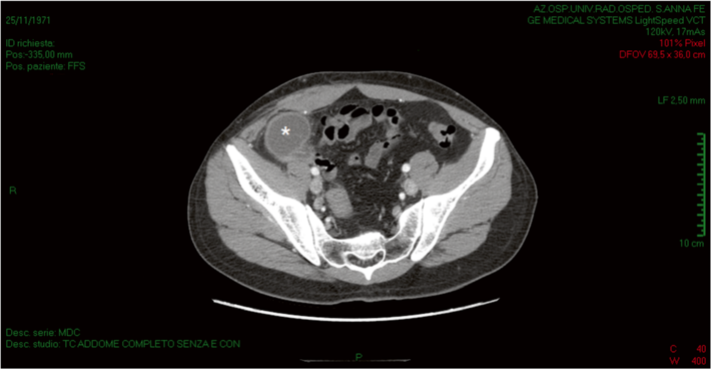
Abdominal computed tomography. Cystic mass (*asterisk*) between the cecum and the right parietocolic recess

**Fig. 3:**
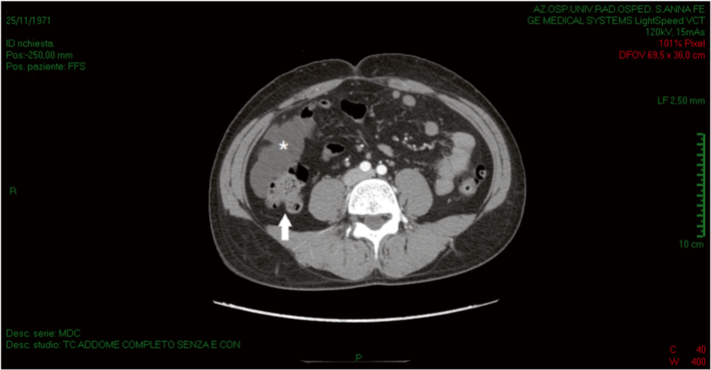
Abdominal computed tomography. Grape-like multicystic mass (*asterisk*) in the right parietocolic recess, adherent to the wall of the ascendant colon (*white arrow*) and parietal peritoneum

Because the evidence from our clinical examination and ultrasonography was unclear, an abdominal CT was performed, which confirmed the presence of an oval formation of 61×42 mm with fluid-overfluid content in direct contiguity with cecal fundus and fluid effusion in pouch of Douglas and along the right parietocolic recess until the punta hepatis (Figs. 2 and 3). Some subcentrimetic lymph nodes near the mass were signaled.

The patient then underwent a further abdominal echography with contrast mean and aspiration of some cloudy dark fluid from which a specimen was taken for a culture and for chemical, physical, and cytological examination. The examinations showed that the fluid was an exudative inflammatory effusion without any significant cytological alteration.

**Fig. 4:**
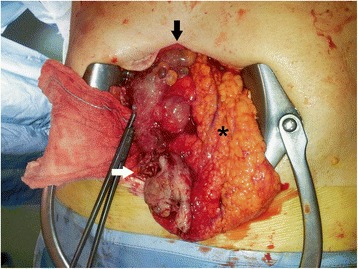
The grape-like multicystic formation (*black arrow*) with necrotic perforated wall (*white arrow*) on the omentum (*asterisk*)

**Fig. 5:**
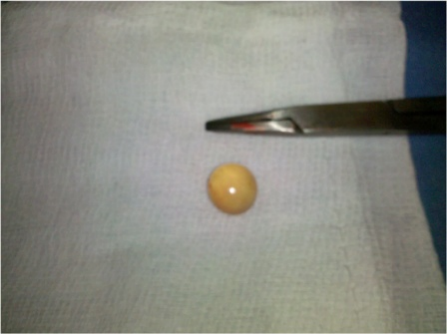
Appearance of the small jelly-like cysts disseminated in the abdomen

Three days after admission, the patient underwent surgery because of the persistence of symptoms and the lack of a definite diagnosis. An explorative laparotomy was performed. At the access to his peritoneal cavity we found conspicuous serohematic effusion and the presence of a grape-like multicystic mass of gelatinous consistence, localized between his cecum and right parietocolic recess, adherent to the wall of his cecum and proximal ascendant colon and parietal peritoneum. On his omentum we found another similar formation that appeared as a cyst with a necrotic perforated wall (Fig. 4). This lesion was first removed and an intraoperative pathological examination was requested: it excluded the presence of neoplastic cells in a pseudocystic formation with fibrous wall, chronic inflammatory infiltration and stratification of fibrinous material above inner surface. We performed an appendicectomy and complete excision of the mass, with cauterization of the abdominal wall to which the neoplasm was adherent, abundant douching of the peritoneal cavity and the removal of all small jelly-like visible cysts disseminated in his abdomen (Fig. 5). No other macroscopic pathological lesions to the explored organs were found.

**Fig. 6:**
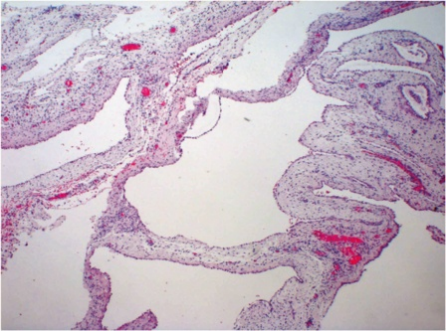
Multiple cysts of varying size with thin walls lined by flattened mesothelial cells (Ematossilina-Eosina (EE); 2×)

Definitive histological diagnosis demonstrated the presence of cystic formations, lined by mesothelium, with thin fibrous wall constituted of tissue of granulation with inflammatory infiltration, compatible with benign peritoneal multicystic mesothelioma (Fig. 6). The omental mass was of the same nature, with evidence of limited adenomatoid areas. Acute catarrhal appendicitis was confirmed with fibrino-leucocytic perivisceral inflammation.

No relevant complications occurred during the postoperative period and the patient was discharged home 8 days after surgery. One year after surgery no evidence of recurrence or residual pathology was observed.

## Discussion

Benign multicystic peritoneal mesothelioma (BMPM) is a rare pathology arising from mesothelial cells of the peritoneal serosa. This lesion usually affects women of reproductive age, but cases in men and children are reported too [2, 3].

The etiology and pathogenesis are unclear. Three hypotheses have been proposed [4, 5]. Some authors consider that BMPM derives from chronic inflammatory processes involving the peritoneum so causing reactive hyperplastic and dysplastic transformation of the mesothelial cells. This theory seems to be sustained by evidence of association of BMPM with some inflammatory *noxae* as previous abdominal surgery or gynecologic diseases (endometriosis, uterine leiomyomas, and pelvic inflammatory disease). Other authors suggest a primitive neoplastic lesion of peritoneal serosa, without strict association with coexistent chronic inflammatory insult. No correlation with asbestos exposure exists. According to a hormonal hypothesis, instead, BMPM development and progression is linked to its sex hormone sensitivity. This theory seems to be supported by the evidence of higher incidence in reproductive-aged women than in men and children, the rare insurgence after annessiectomy and in menopause, and the responsiveness to treatment with tamoxifen and gonadotropin-releasing hormone analog agonists [6].

BMPM can arise in the whole peritoneum, but the most frequent site affected is the pelvis [7].

Classically, the lesion presents as a thin-walled cystic structure that can consist in a single unilocular cyst or in a grape-like cluster of cysts of variable sizes, lined by flat or cuboidal mesothelium-like epithelium and containing serous fluid. Inflammatory cells and fibrous elements can be found within the stroma between the cysts and foci of mesothelial hyperplasia may also be present [8].

There are problems of differential diagnosis with benign and malignant conditions such as lymphangioma, mesenteric or omental cysts, visceral cysts, echinococcal cysts, cystic adenomatoid tumors, malignant mesothelioma, cystic mesonephric duct remnants, endometriosis, endosalpingiosis, hydrosalpinx, tubo-ovarian abscess, ovarian cystadenoma or cystadenocarcinoma, cystic teratoma, pseudomyxoma peritonei, cystic smooth muscle tumors, cystic mucinous neoplasm of the pancreas, and non-pancreatic pseudocysts [8, 9]. Lymphangioma is usually a large multiloculated cystic mass containing chylous fluid and bounds of smooth muscle and aggregates of lymphocytes in its wall. Mesenteric cysts are generally unilocular and contain serous secretions. A cystic variant of adenomatoid tumors often presents a recognizable solid component. Malignant mesothelioma usually affects older patients with a history of previous asbestos exposure; it presents as multiple small nodules besides cystic structures and shows malignant features such as infiltrative growth and cellular atypias. A teratoma often contains fat and calcifications. In cases of pseudomyxoma peritonei, generally soft-tissue peritoneal nodules and omental caking are present. Malignant neoplasms are usually associated with intramural nodules, ascites, necrosis or peritoneal carcinomatosis and the source organ can usually be identified [8].

Cystic mesothelioma is traditionally considered to be a benign neoplasm, but some authors underline the potential malignant behavior of this neoplasm, linked to the high local recurrence rate (about 50 %) observed after complete surgical resection [10].

Clinical presentation of BMPM is generally aspecific. The lesion can sometimes be asymptomatic until the accidental diagnosis. The most common presenting symptoms are chronic or intermittent abdominal or pelvic pain, tenderness and distension, due to a palpable and painful mass or to mechanical compression of adjacent structures and organs. An association between BMPM and increased serum carbohydrate antigen 19–9 (CA 19–9) concentration has been described. In our case all tested neoplastic markers were negative: carcinoembryonic antigen (CEA), CA 19–9, and alpha-fetoprotein. Ultrasonography, CT scan and magnetic resonance imaging (MRI) are helpful to evaluate the localization and the size of the neoplasm, but they do not enable diagnosis of BMPM or the exclusion of other cystic lesions of the peritoneum. Laparoscopy can be considered to perform biopsies for histologic diagnosis or for surgical treatment [11, 12].

Acute presentation is uncommon and the association with acute appendicitis, as in our case, is extremely rare, so only few cases are reported in the literature [1, 13].

There are no evidence-based strategies of treatment for BMPM. Complete surgical removal has been advocated by most authors as the treatment of choice, but considering the high rate of recurrence and the borderline behavior of these neoplasms more aggressive approaches have been recently proposed, such as cytoreductive surgery with standard peritonectomy procedures in combination with hyperthermic intraperitoneal chemotherapy (HIPEC) [14, 15]. Some studies suggest that cytoreductive surgery and HIPEC can provide better disease control than debulking surgery alone, considering the high risk of recurrence and the possibility of malignant transformation of this borderline neoplasm in a patient inadequately treated. Because comparative studies are infeasible, there is no census on whether debulking surgery should be considered the first-line treatment with cytoreductive surgery with HIPEC reserved as a second-line option [16, 17].

## Conclusions

We reported the uncommon case of an acute presentation of a rare benign pathology that is peritoneal multicystic mesothelioma. We underlined the difficulties that a similar presentation poses in terms of differential diagnosis and therapeutic strategy: in fact, images can provide suspicion of this kind of neoplasm, but definitive diagnosis is done only by histological examination, and there are no well-defined and evidence-based treatments.

## Consent

Written informed consent was obtained from the patient for publication of this case report and any accompanying images. A copy of the written consent is available for review by the Editor-in-Chief of this journal.

## Abbreviations

BMPM: Benign multicystic peritoneal mesothelioma
CA 19–9: Carbohydrate antigen 19–9
CEA: Carcinoembryonic antigen
CT: Computed tomography
HIPEC: Hyperthermic intraperitoneal chemotherapy
MRI: Magnetic resonance imaging
